# High-Throughput Carrier Screening Using TaqMan Allelic Discrimination

**DOI:** 10.1371/journal.pone.0059722

**Published:** 2013-03-26

**Authors:** Anastasia Fedick, Jing Su, Chaim Jalas, Lesley Northrop, Batsal Devkota, Josef Ekstein, Nathan R. Treff

**Affiliations:** 1 Department of Microbiology and Molecular Genetics, University of Medicine and Dentistry of New Jersey-Robert Wood Johnson Medical School, Piscataway, New Jersey, United States of America; 2 Reproductive Medicine Associates of New Jersey, Basking Ridge, New Jersey, United States of America; 3 Bonei Olam, Center for Rare Jewish Genetic Disorders, Brooklyn, New York, United States of America; 4 Dor Yeshorim, The Committee for Prevention of Jewish Diseases, Brooklyn, New York, United States of America; Tor Vergata University of Rome, Italy

## Abstract

Members of the Ashkenazi Jewish community are at an increased risk for inheritance of numerous genetic diseases such that carrier screening is medically recommended. This paper describes the development and evaluation of 30 TaqMan allelic discrimination qPCR assays for 29 mutations on 2 different high-throughput platforms. Four of these mutations are in the *GBA* gene and are successfully examined using short amplicons due to the qualitative nature of TaqMan allelic discrimination. Two systems were tested for their reliability (call rate) and consistency with previous diagnoses (diagnostic accuracy) indicating a call rate of 99.04% and a diagnostic accuracy of 100% (+/−0.00%) from one platform, and a call rate of 94.66% and a diagnostic accuracy of 93.35% (+/−0.29%) from a second for 9,216 genotypes. Results for mutations tested at the expected carrier frequency indicated a call rate of 97.87% and a diagnostic accuracy of 99.96% (+/−0.05%). This study demonstrated the ability of a high throughput qPCR methodology to accurately and reliably genotype 29 mutations in parallel. The universally applicable nature of this technology provides an opportunity to increase the number of mutations that can be screened simultaneously, and reduce the cost and turnaround time for accommodating newly identified and clinically relevant mutations.

## Introduction

The high prevalence of carriers of recessive mutations in the Ashkenazi Jewish population [1] has made genotyping and carrier screening imperative. Carrier screening was first introduced for Tay-Sachs disease in the early 1970s, and because of the positive effect it had in reducing the number of children conceived with the disease, screening for Cystic Fibrosis and Gaucher Disease began in 1993 [2], [3]. Since that time, the American College of Medical Genetics (ACMG) has also advised testing for six additional diseases including Bloom Syndrome, Canavan disease, Familial dysautonomia, Fanconi anemia group C, Mucolipidosis IV, and Niemann-Pick disease type A [4], [5]. This has led to an increasing demand for quick and accurate diagnoses, and advances in detecting mutations and determining genotypes [6], [7]. Methods such as Restriction Fragment Length Polymorphism (RFLP) testing [8] or the Amplification Refractory Mutation System (ARMS) [9], however, are hindered by time-consuming gel-based evaluation of PCR amplicons. Additionally, the platforms that have been developed for high-throughput genotyping purposes, such as a PCR and matrix-assisted, laser desorption/ionization time of flight (MALDI-TOF) mass spectrometry method [10] or arrayed primer extension (APEX) [11], are labor-intensive. Therefore, a more efficient, high-throughput, and automated method of screening for common mutations is needed.

Real time PCR with TaqMan allelic discrimination has frequently been used to characterize single nucleotide polymorphisms (SNPs) [12]–[14] and an insertion/deletion (indel) polymorphism 276 base pairs in length [15]. It has also recently been shown to allow for the detection of large deletions [16] using the same methodology as applied to genotyping SNPs and small indels, and TaqMan Reverse transcription-PCR has been used to make clinical diagnoses for viral infections [17]. The combined PCR and allelic discrimination procedure can be performed in a highly parallel manner using new technologies which allow nanoliter reactions to be performed. In addition, automated sample preparation, reaction setup, and data analysis, make this method ideal for genotyping a broad range of mutations in a high-throughput fashion.

In this paper, 30 individual TaqMan genotyping assays were designed for a subset of the ACMG approved mutations (ranging from point mutations to small and large indels) responsible for causing Bloom Syndrome, Canavan disease, Cystic fibrosis, Familial dysautonomia, Fanconi Anemia Type C, Gaucher disease, Mucolipidosis IV, Niemann-Pick disease, and Tay-Sachs disease. The four TaqMan assays designed to detect the Gaucher's mutations targeted short amplicons, consistent with TaqMan requirements, despite the similarities of the target *GBA* gene to the pseudogene, *GBAP*. All of the assays were validated on control samples and then evaluated on 2 platforms for high-throughput genotyping. While the mutations tested here represent those prevalent in the Ashkenazi Jewish population, the same methodology can be used to design TaqMan assays for other mutations.

## Materials and Methods

### Ethics Statement

All research material was obtained through written patient consent. Institutional review board permission was not required due to the removal of all sample identifiers prior to receipt by our lab (45 CFR part 46.101(b)(4)).

### Experimental Design

This study was conducted in multiple phases. TaqMan assays were designed and validated on genomic (gDNA) with previously characterized genotypes for the targeted mutations. Three large blind validations were then done on different sets of gDNA. The first blind analysis was performed using conventional methods for qPCR (large volume reactions), the second blind analysis was done on two high-throughput platforms to assess applicability, accuracy (consistency), and reliability of genotyping in a manner consistent with routine application, and the third was done to test assay performance for several mutations at their expected carrier frequencies.

### Population

The gDNA used in this study was obtained from individuals of Ashkenazi Jewish decent. The gDNA was extracted from blood using the QIAamp DNA Blood Maxi Kit or from buccal swabs using the Gentra Puregene Buccal Cell Kit, (QIAGEN Inc, Germantown, MD, USA) and the concentrations were obtained via Nanodrop (Thermo Fisher Scientific Inc., Wilmington, DE, USA).

### Assay Design

The assays were designed by using NCBI to search for the full sequence of the gene (FASTA). Once the gene was found, roughly two hundred base pairs upstream and downstream of the mutation site were selected and put into Repeat Masker (Institute for Systems Biology, Seattle, WA, USA) to mask for the repeats. The original selected sequence was then put into the NCBI Blast site (SNP Flanks) so that SNPs could be masked. The assays were then made in File Builder software (Life Technologies [LTI], Carlsbad, CA, USA) or designed with Primer Express Software (LTI). The assays targeted ten missense, four nonsense, three frameshift, eight splicing, three small deletions, and one large deletion (6,433 base pair) mutation. The assay design for the large deletion has been previously published [16] and required the use of two assays. The context sequences for all of the assays are available in Table S1 as in compliance with the minimum information for the publication of real-time quantitative PCR experiments (MIQE) guidelines [18]. All of the assays were designed so that the wild type allele utilized the VIC probe and the minor allele utilized the FAM probe.

### Assay Validation

To validate the assays, known heterozygous carrier and/or homozygous affected control gDNA samples were normalized to 5 ng/uL. The number of samples tested for each assay varied based on the availability of the gDNA, but always included one wild type sample and a no template control (NTC). The gDNA was plated in duplicate (to ensure accurate genotyping) in 384 well plates along with TaqMan Master Mix (LTI) and the assay bringing the final volume to 5 uL. The plates were centrifuged for 1 minute, sealed, and then run in duplex real time PCR reactions using FAM and VIC as the detector probes for each assay on both the ABI PRISM® 7900 HT Sequence Detection System (LTI) and the Applied Biosystems ViiA™ 7 Real-Time PCR System (LTI). After the real time PCR was finished, allelic discrimination analysis was performed using SDS 2.3 software (LTI).

A blinded study was then performed on the ABI PRISM® 7900 HT Sequence Detection System. Every assay was tested on 382 blind samples that were normalized to 2 ng/uL and 2 NTCs. The samples were plated in duplicate in 384 well plates at a volume of 5 uL, dried, and then TaqMan Master Mix and assay (premixed) were added for a final volume of 5 uL. The plates were centrifuged for 1 minute, sealed, and then run in duplex real time PCR reactions using FAM and VIC as the detector probes for each assay. Allelic discrimination analysis was performed using SDS 2.3 software and the data was analyzed in TaqMan Genotyper v1.1 (LTI).

Assay performance was then tested on the OpenArray® Real-Time PCR Platform (LTI) and the Fluidigm BioMark™ HD System (Fluidigm Corporation [FC], San Francisco, CA, USA). 470 samples with a variable number of carriers for each mutation (Table 1) and 10 NTCs were normalized to 50 ng/uL. For the OpenArray® Real-Time PCR Platform, 2.5 uL of the TaqMan® OpenArray® Genotyping Master Mix (LTI) and 2.5 uL of each gDNA sample were premixed in a 384 well plate and transferred to the genotyping plates using the OpenArray AutoLoader. For the Fluidigm BioMark™ HD System, the samples were premixed with master mix for a final volume of 5 uL and both the samples and assays were pressure loaded into reaction chambers using the IFC Controller. 95% confidence intervals were included in the calculations for accuracy, diagnostic accuracy, sensitivity, and specificity for both platforms. To calculate reproducibility, the coefficient of variation was calculated for the VIC and FAM signals for the wild type samples of every assay on both platforms. Notches equivalent to 95% confidence intervals [19] were calculated for the boxplot based on the formula: +/−1.58 IQR/sqrt(n).

**Table 1:** Mutations detected by TaqMan assays.

Disease	Gene	Mutation	No. Carrier Samples*	No. Affected Samples*
Bloom Syndrome	BLM	c.2207_2212delinsTAGATTC	11	1
Canavan	ASPA	c.914C>A	1	0
Canavan	ASPA	c.854A>C	25	0
Canavan	ASPA	c.693C>A	9	0
Cystic Fibrosis	CFTR	c.3454G>C	8	0
Cystic Fibrosis	CFTR	c.3909C>G	5	0
Cystic Fibrosis	CFTR	c.3276C*>*A	1	0
Cystic Fibrosis	CFTR	c.3846G>A	35	1
Cystic Fibrosis	CFTR	c.1624G>T	8	1
Cystic Fibrosis	CFTR	c.1521_1523delCTT	35	0
Cystic Fibrosis	CFTR	c.3718−2477C>T	6	0
Cystic Fibrosis	CFTR	c.1585−1G>A	3	0
Cystic Fibrosis	CFTR	c.2988+1G>A	1	0
Familial dysautonomia	IKBKAP	c.2087G>C	4	0
Familial dysautonomia	IKBKAP	c.2204+6T>C	43	1
Fanconi Anemia Type C	FACC	c.456+4A>T	26	0
Gaucher Disease	GBA	c.84_85insG	6	0
Gaucher Disease	GBA	c.1226A>G	43	0
Gaucher Disease	GBA	c.1448T>C	3	0
Gaucher Disease	GBA	c.115+1G >A	4	0
Neimann-Pick type A	SMPD1	c.1493G>T	8	1
Neimann-Pick type A	SMPD1	c.911T>C	7	0
Neimann-Pick type A	SMPD1	c.996delC	12	0
Neimann-Pick type B	SMPD1	c.1829_1831delGCC	10	0
Tay-Sachs	HEXA	c.1274_1277dupTATC	37	1
Tay-Sachs	HEXA	c.805G>A	3	0
Tay-Sachs	HEXA	c.1421+1G>C	19	0
Mucolipidosis IV	MCOLN1	c.406−2A>G	17	1
Mucolipidosis IV	MCOLN1	g.511_6943del	20	0

* Note: Samples run in duplicate during experiments

The additional mutations tested at their expected carrier frequencies were run on the QuantStudio™12K Flex Real-Time PCR System (LTI). 192 samples were normalized to 50 ng/uL. 2.5 uL of TaqMan® OpenArray® Genotyping Master Mix (LTI) and 2.5 uL of each gDNA sample were premixed in a 384 well plate and loaded onto the genotyping plates using the QuantStudio™ 12K Flex OpenArray® AccuFill™ System. The results were analyzed in TaqMan Genotyper v1.2 (LTI).

### NGS Data Acquisition and Analysis

Nine gDNA samples were normalized to 5 ng/uL and amplified for 14 cycles of PCR using the four *GBA* TaqMan assays and PreAmp Master Mix as recommended by the supplier (LTI). The Ion Xpress™ Plus gDNA and Amplicon Library Preparation protocol was used for the barcoded, short amplicons procedure (LTI). Original concentrations were obtained using a Nanodrop-8000 spectrophotometer, and molar concentrations were obtained using a Bioanalyzer on the Agilent High Sensitivity DNA microfluidic chip (Agilent Technologies Inc, Santa Clara, CA). The Ion OneTouch Template Kit was used for template preparation and the Ion Sequencing Kit v2.0 was used for the Ion 316 Chip-based sequencing (LTI).

FASTQ files were then obtained from the Ion Torrent Server and aligned against the reference sequence, which consisted of the nucleotides in the four target *GBA* amplicons, using Bowtie 2. Local alignment was done with default parameters to output the alignment file in Sequence Alignment/Map (SAM) format. These files were then converted to BAM (binary version of SAM) format using SAMtools. The BAM files were loaded in the Integrative Genomic Viewer (IGV) from Broad Institute so sequence alignment could be observed. Aligned reads with the reference sequence were displayed in the IGV interface and the count of each nucleotide as it corresponded to the reference sequence for each position, based on the total number of reads at that particular position, was obtained.

## Results

**Figure 1:**
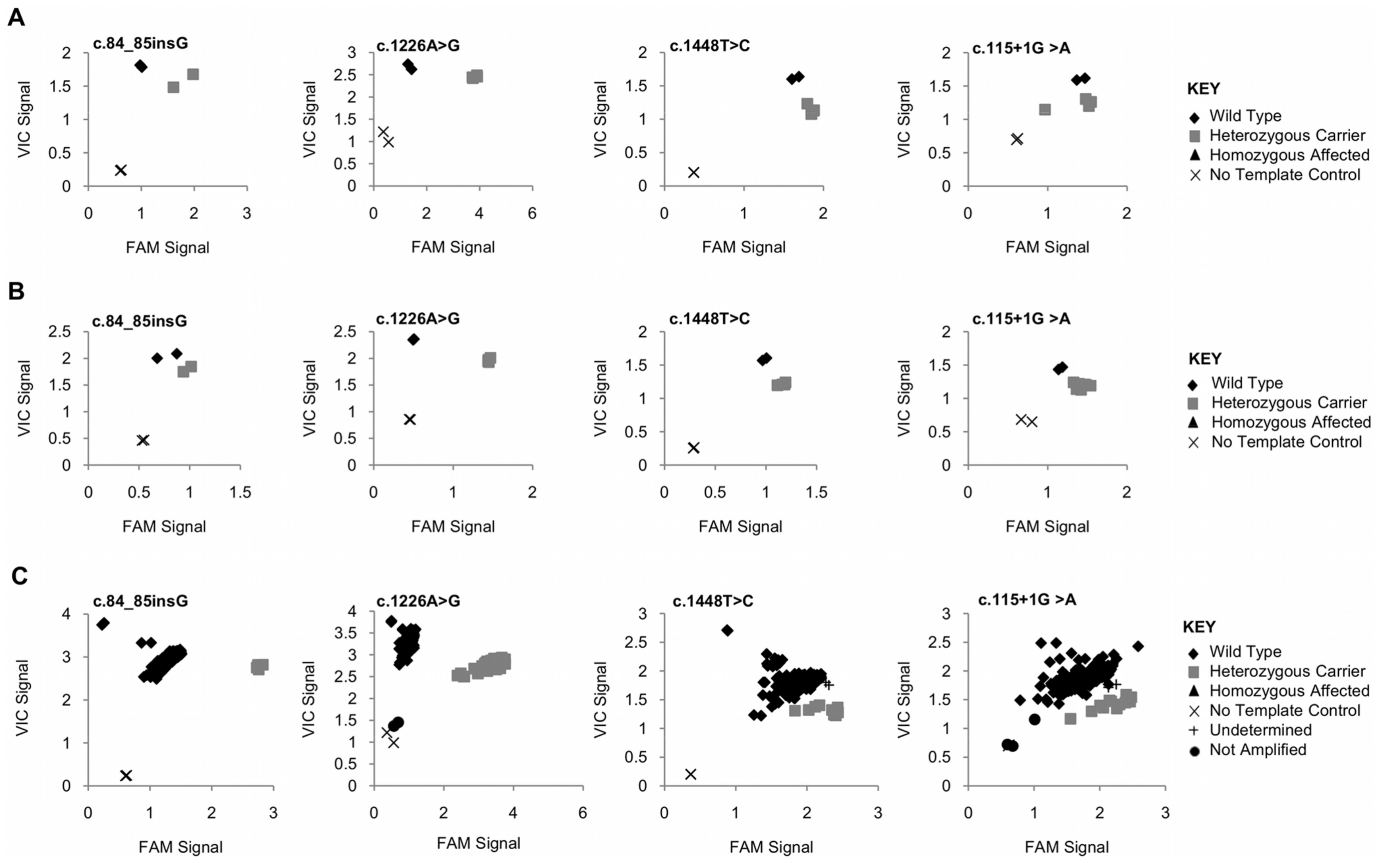
Platform comparison. Allelic discrimination plots of the four *GBA* mutations. Water was used as the no template control. Fig. 1A: Validation on the ABI PRISM® 7900 HT Sequence Detection System. Fig. 1B: Validation on the Applied Biosystems ViiA™ 7 Real-Time PCR System. Fig. 1C: Blind validation on the ABI PRISM® 7900 HT Sequence Detection System.

**Figure 2:**
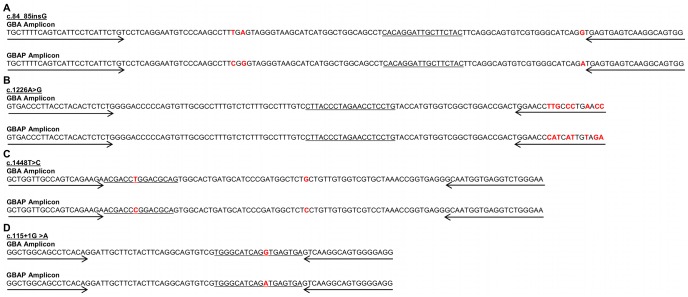
Sequence differences for *GBA* and *GBAP* amplicons. Nucleotide sequences of the target amplicons in both the *GBA* and *GBAP* genes highlighting both the similarities and differences between the sequences. Forward and reverse primers are indicated by arrows and the probe is underlined. Nucleotide differences are indicated by red, bold lettering.

The initial validation of the assays on a small scale yielded 100% genotyping accuracy. Figure 1 shows the successful genotyping of the 4 *GBA* mutations on both the ABI PRISM® 7900 HT Sequence Detection System and the Applied Biosystems ViiA™ 7 Real-Time PCR System (see Figures S1 and S2 for other assay results). Unique clusters indicative of different genotypes were formed based on the signal intensity ratio of the two probes being used (VIC and FAM), predicting the genotypes with an accuracy of 100%. Because TaqMan allelic discrimination is a qualitative calculation, the similarities of the four *GBA* target sequences to the pseudogene did not affect the clustering other than uniformly shifting all of the clusters either towards the VIC or FAM axis based upon which probe was naturally also present in the *GBAP* gene. As seen in Figure 2, based on the nucleotides in the primer sequences, three of the four *GBA* assays would amplify sequences in both the *GBA* and *GBAP* genes. Additionally, the mutant probes for two of the *GBA* mutations had the same sequence as the wild type probes for *GBAP*, and the same wild type probe was present for the c.84_85insG mutation in both genes.

The non-specificity of the primers was confirmed through next generation sequencing (NGS) using the Ion Torrent Personal Genome Machine (PGM). NGS generates highly parallel data at base pair resolution, and allows multiple changes in a single amplicon to be observed in one viewing. Since each amplicon had at least one nucleotide that was different between the *GBA* and *GBAP* genes, the percentage that each gene was being targeted by the TaqMan assays could be determined by looking at these specific alleles. Results for two samples sequenced for this purpose are shown in Table 2, confirming the non-specificity of the primers for 3 of the 4 mutations. This non-specificity did not affect the genotyping calls, however, indicating that (q)PCR and allelic discrimination of short amplicons can successfully provide genotype results for *GBA* mutations.

**Table 2:** Percentage each gene is targeted based on informative SNPs.

	Sample 1		Sample 2	
Mutation	GBA	GBAP	GBA	GBAP
c.84_85insG	58%	42%	59%	41%
c.1226A>G	99%	0%	99%	0%
c.1448T>C	39%	61%	32%	70%
c.115+1G >A	47%	53%	51%	49%

* Note: For replicates to fail, both data points had to have the wrong genotype assigned

**Figure 3:**
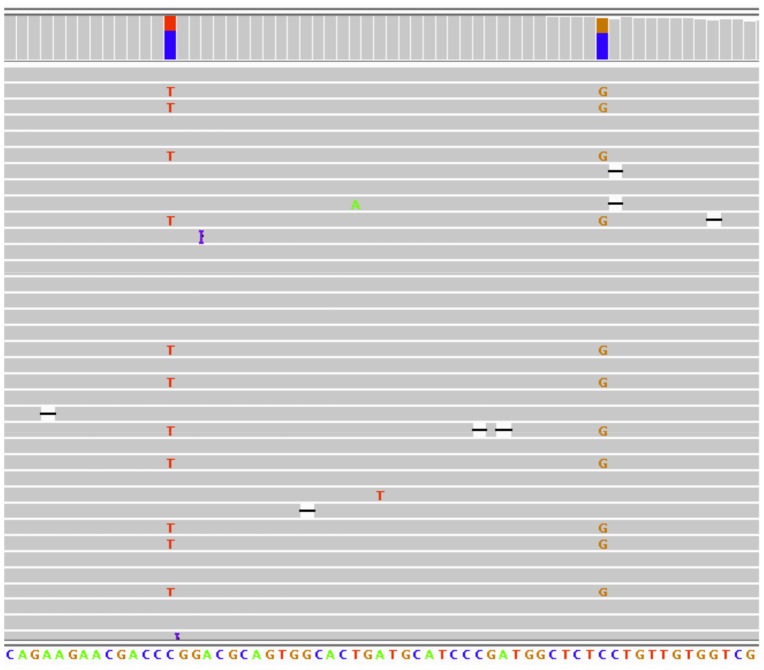
NGS of c.1448T>C mutation. NGS integrative genomics viewer plot of a sample wild type for the c.1448T>C mutation. The plot includes a vertical bar graph (columns on top) which indicates the depth at each base, and in this situation, that both the *GBA* and *GBAP* genes are being targeted. Letter codes for each position are indicated at the bottom and represent the *GBAP* human genome reference sequence. Each plot also contains multiple horizontal bars representing individual sequence reads (grey bars), with a purple symbol indicating an insertion, a black dashed line indicating a deletion, and a letter indicating a variant relative to the reference sequence. For this mutation, a T allele at the mutation sight and a G allele at the informative SNP site indicates the wild type *GBA* gene, a C allele at the mutation site and a G allele at the informative SNP site indicates the mutated *GBA* gene, and a C allele at both the mutation and informative SNP site indicates the *GBAP* gene.

To further investigate the accuracy of genotyping *GBA* mutations with short amplicons, 87 blind samples and an NTC were plated in duplicate and genotyped on the ABI PRISM® 7900 HT Sequence Detection System for the c.1448T>C mutation. Results were 100% concordant with those obtained using restriction enzymes highly specific to the normal copy of the gene, and indicated that 9 samples were wild type and 78 were carriers. To further confirm the genotypes, NGS was done for the wild type samples using the SNPs that differed between the *GBA* and *GBAP* sequences to determine which gene was being targeted and at what percentage. As indicated in Figure 3, using these informative SNPs in combination with parallel sequencing allowed for the successful detection of genotypes despite the assays having targeted both genes. When viewing the results, if there was a T allele present at the mutation site and a G allele present at the informative SNP site on the same strand, then the *GBA* gene had been targeted and the sequence was wild type. Similarly, if there was a C allele at the mutation site and a G allele at the informative SNP site, the *GBA* gene was still being targeted, but the sequence was the mutation. If there was a C allele at the mutation site and a C allele at the informative SNP site, then the *GBAP* gene had been targeted. By looking at the ratios of alleles at the mutation site for the *GBA* gene and ignoring the *GBAP* sequences, the genotypes of the samples could be obtained. For instance, the sample in Figure 3 was wild type because the frequency of the T and G allele pair was ∼50%, and the C and C allele pair was ∼50%. If the sample was a carrier, the T and G allele pair would be ∼25%, the C and G allele pair would be ∼25%, and the C and C allele pair would be ∼50%. NGS confirmed the TaqMan-assigned genotypes for all nine samples. Additionally, in no instance was a conversion of the pseudogene detected, indicating that this method can be used to genotype GBA mutations.

A large blind validation for all of the assays was then done, and indicated that 27 of the 30 assays had an autocalled accuracy of 100% (Figure 1c and Figure S3). The sample results were autocalled by TaqMan Genotyper v1.1 software and then manually reviewed. The genotyping clusters of two of the splice site mutations, (c.2204+6T>C in the *IKBKAP* gene and c.1421+1G>C in the *HEXA* gene), were clustered too closely for the software to make automatic calls. Manual calls were made with an accuracy of 100%, however, indicating that the two assays did not need to be redesigned. The assay designed to detect the indel in the *BLM* gene, however, had to be redesigned because the genotyping clusters were too close for automatic or manual calls to be made. Figure S4 shows both the original and revised assay results as tested on control samples. After redesigning the assay, the separation of the genotyping clusters increased significantly allowing automatic calls to be made.

The blind validation also included determining if the presence of other SNPs in the area of the target mutation would negatively affect genotypes obtained using TaqMan assays. The c.1521_1523delCTT (F508) mutation was specifically investigated because of the known benign SNPs located near this small deletion [20]. 200 different blind DNA samples were genotyped for this mutation using the TaqMan assay in this panel. All of the samples were genotyped as being heterozygous carriers, which was concordant to their original genotypes. These results demonstrate that in this specific population, the performance of the F508 TaqMan assay was not negatively affected by theoretical nearby SNPs.

The assays were then tested for high-throughput genotyping by transitioning from 384-well plates to the OpenArray® Real-Time PCR Platform (LTI) and the Fluidigm BioMark™ HD System (FC). After the analysis was complete, the observed genotypes were compared to the expected genotypes of the control samples. The OpenArray® Real-Time PCR Platform had an individual data point call rate of 99.04%, a replicate call rate of 98.09%, an individual data point accuracy of 99.89% (+/−0.12%), and a diagnostic accuracy of 100% (+/−0.00%) (See Table 3). Samples that had different genotypes assigned between the two replicates were considered undetermined and rerun on the ABI PRISM® 7900 HT Sequence Detection System. The Fluidigm BioMark™ HD System had an individual data point call rate of 94.66%, a replicate call rate of 89.32% an individual data point accuracy of 92.96% (+/−0.39%), and a diagnostic accuracy of 93.35% (+/−0.29%). Additionally, the OpenArray® Real-Time PCR Platform had a sensitivity of 98.82% (+/−0.87%) and a specificity of 99.95% (+/−0.15%), while the Fluidigm BioMark™ HD System had a sensitivity of 94.89% (+/−0.02%) and a specificity of 92.91% (+/−0.12%).

**Table 3:** Platform Comparison Results.

	Total Data Pts	Data Pt SNP Call	SNP Call Rate	Replicate SNP Call Rate	Accurate SNP Call	Accuracy	No. Failed Replicates*	Diagnostic Accuracy	Unamplified Data Pts	Failed Wells
Open Array	28200	27930	99.04%	98.09%	27898	99.89%	0	100%	156	4
Fluidigm	28200	26694	94.66%	89.32%	24814	92.96%	938	93.35%	1146	360

The performance discrepancy between the two systems stemmed largely from the Fluidigm BioMark's inability to genotype two mutations occurring in the *GBA* gene (c.115+1G >A and c.1448T>C). For both mutations, only one genotyping cluster formed, which was autocalled as the carrier genotype. The probe sequence for the mutation in the *GBA* gene was the same as the wild type probe for the *GBAP* gene in both of these mutations, meaning that the clusters were shifted along the FAM axis. It is therefore possible that this shift caused a problem for genotyping on the Fluidigm BioMark. The assays performed accurately on the OpenArray system, however, suggesting that the designs are sufficient for high-throughput genotyping on some platforms. In order to perform better on the Fluidigm BioMark, the assays could potentially be redesigned with the primers covering one of the nucleotide bases that are variable between the two sequences.

**Figure 4:**
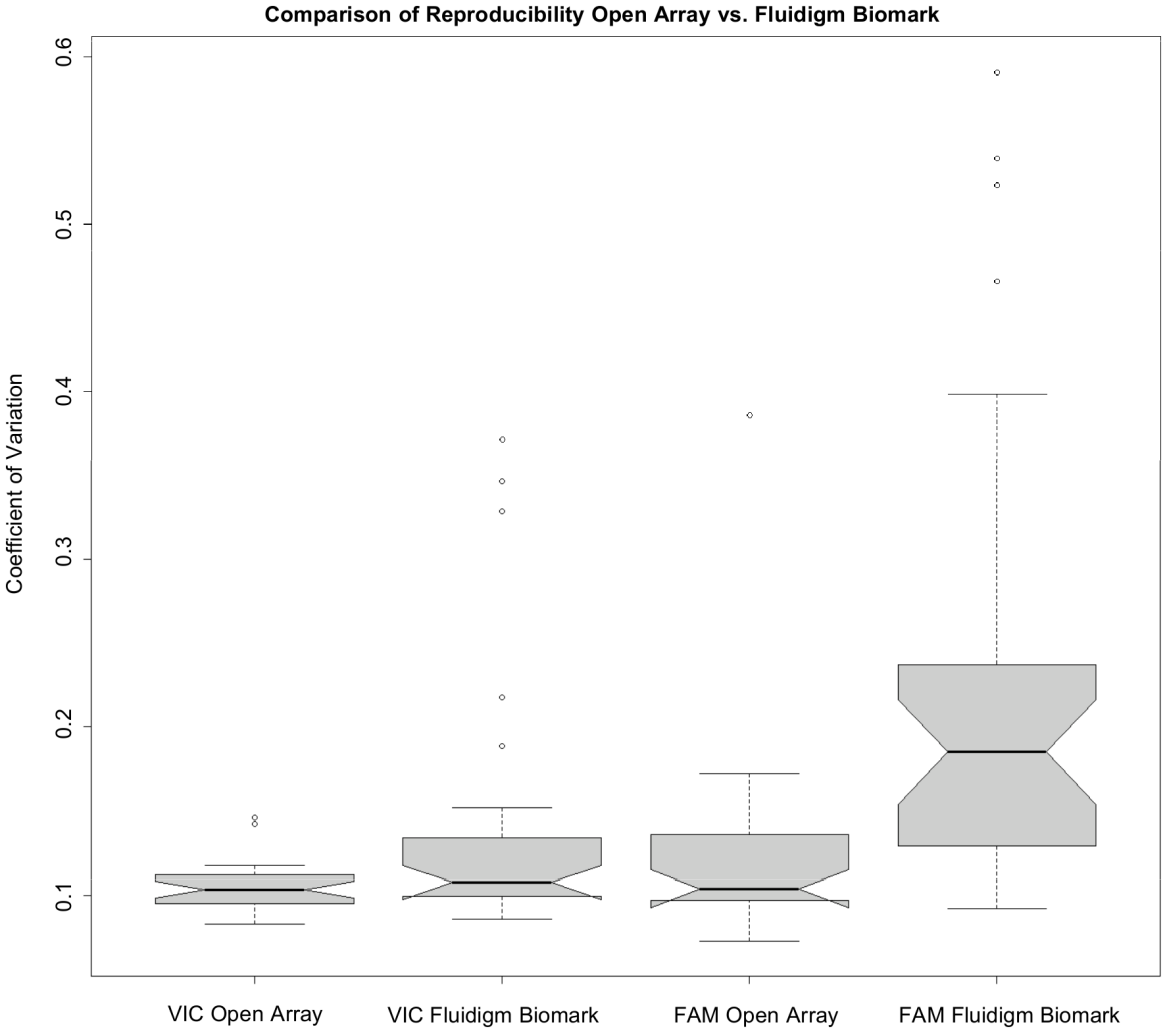
Platform comparison for reproducibility. A notched box plot depicting the coefficient of variation for the FAM and VIC probes for the OpenArray® Real-Time PCR Platform and Fluidigm BioMark™ HD System. The coefficient of variation was calculated for the expected wild type samples, regardless of the final calls made by the instruments, for all 30 assays. The same samples were compared between instruments per assay, and the same numbers of plates were run on each instrument. The notches in the boxplot represent a 95% confidence interval.

The reproducibility of the platforms was evaluated by calculating the coefficient of variation (the standard deviation divided by the mean) for the VIC and FAM signals for the expected wild type samples of every assay on both platforms. The same samples were compared for each assay on each platform, and plate effects were avoided by using the same number of plates on each instrument. A two-sided, paired *t*- test with a 95% confidence interval showed that the Fluidigm BioMark had more variation in both the VIC and FAM signals when compared to the OpenArray. The p-value for the VIC signal was significant at 0.01814 and the p-value for the FAM signal was very significant at 0.000684. Figure 4 shows a notched box plot comparing the reproducibility between the two platforms, where the notches are equivalent to 95% confidence intervals. The notches for the two different FAM signals do not overlap, which indicates that the difference between the medians is statistically significant. The difference between the VIC signals is less pronounced, consistent with the *t-*test results. As previously stated, however, the Fluidigm Biomark's inability to genotype two mutations is influencing this result.

Of the 29 mutations examined, 4 were originally tested at their expected carrier frequencies (*ASPA* c.914C>A, *ASPA* c.693C>A, *CFTR* c.3276C*>*A, and *SMPD1* c.911T>C) [21], [22]. These assays had an individual call rate of 99.28%, a replicate call rate of 99.04%, and an individual data point accuracy, a diagnostic accuracy, a sensitivity, and a specificity all of 100% on the OpenArray® Real-Time PCR Platform. Similarly, these assays had an individual call rate of 98.16%, a replicate call rate of 96.33%, and an individual data point accuracy, a diagnostic accuracy, a sensitivity, and a specificity all of 100% on the Fluidigm BioMark™ HD System. To further explore this, a second blind experiment was done on the OpenArray for four additional mutations (*ASPA* c.854A>C, *CFTR* c.3846G>A, *IKBKAP* c.2204+6T>C, and *MCOLN1* c.406−2A>G) at their respective carrier frequencies as reported in literature [20]. 192 samples were plated in duplicate and the combined results from the two studies yielded an overall individual call rate of 97.87%, a replicate call rate of 96.07%, an accuracy of 99.92% (+/−0.21%), a diagnostic accuracy of 99.96% (+/−0.05%), a sensitivity of 96.43% (+/−0.05%), and a specificity of 99.98% (+/−0.009%). These results therefore indicate that the assays are capable of successfully identifying heterozygous carrier samples at their expected frequencies in the population on a high-throughput platform.

## Discussion

Mutations responsible for causing diseases present at higher frequencies in the Ashkenazi population were quickly and accurately screened for using TaqMan assays and high-throughput genotyping platforms. The diagnostic accuracy of the OpenArray was higher than the Dynamic Array [23] and other methods such as MALDI-TOF mass spectrometry [10]. The overall throughput for the two platforms tested ranges from approximately 55,000–70,000 genotypes per day on one instrument, which is comparable if not higher than other platforms. Additionally, when considering that the large deletion would have previously needed to be genotyped separately through copy number analysis, or multiplex ligation-dependent probe amplification [24], these platforms have increased the efficiency of genotyping.

In order to reduce sample preparation time, the use of the TaqMan® Sample-to-SNP™ Kit (LTI) is currently being investigated. This procedure allows samples to be genotyped directly from blood as opposed to having to isolate the gDNA first. The potential benefits include avoiding purification, thereby reducing expense and giving a more rapid turnaround time, as well as better automation [25]. The incorporation of quality control measures to ensure that no cross contamination occurs is also recommended. In this study the samples were always run in duplicate to ensure that every individual was genotyped correctly by essentially genotyping them twice. The addition of a gender determination assay and/or assays that target specific SNPs for DNA fingerprinting can also be used as patient/sample identification controls. In order to validate a gender assay that uses the single base differences in the amelogenin genes to determine the presence of the X and Y chromosomes [26], we tested the assay on 1,012 samples using the ABI PRISM® 7900 HT Sequence Detection System. Gender results were 100% concordant with expected results, indicating that this assay would be a good candidate to include in the array. The development of multiplexing assays for the deletion mutations is also being investigated so that the use of two assays and two result plots per deletion will not be necessary.

Included in the mutation panel were four assays used to genotype four mutations in the *GBA* gene, which is located on chromosome 1q21 in close proximity to pseudogene *GBAP* [27]. Since the *GBA* gene is 96% homologous to *GBAP*, long amplicons are normally needed to ensure that only the *GBA* gene is being targeted when genotyping [28]. TaqMan genotyping is done with short amplicons, however, and therefore three of the four primer pairs were not specific to the *GBA* gene, amplifying both the *GBA* and the *GBAP* target sequences. The only influence that the pseudogene had, however, was to uniformly shift the genotyping clusters towards either the VIC or FAM axis depending on which probe sequence was naturally present in it. Therefore, even though both genes were being targeted, the assays were still able to reliably genotype samples based on the consistency of the pseudogene sequence. While gene conversion, fusion events, and reciprocal crossing over between the *GBA* and *GBAP* genes are believed to be the cause of mutations in the *GBA* gene, [29]–[31] to our knowledge no mutations have been reported to occur in the *GBAP* gene at the four mutation sites focused on in this paper, and NGS on nine samples showed no evidence of this occurring either. The non-specificity of the primers would only be an issue if there was a mutation in the *GBAP* gene at the exact site where the mutation was in the *GBA* gene, and the allele change matched the alleles present in the *GBA* gene. In the event of this occurring, it would not be possible to reliably genotype the mutation using this method.

Further investigation into using TaqMan genotyping assays in combination with available high-throughput genotyping platforms will be carried out by our lab in order to improve the accuracy of the tests for clinical use. The use of these assays can be beneficial not only for carrier screening, but also for newborn screening using genotyping and for preimplantation genetic diagnosis (PGD) of embryos undergoing *in vitro* fertilization. Furthermore, these assays can be used on genotyping platforms for qPCR or allelic discrimination, or be used to perform targeted NGS. Targeted NGS has recently been shown to provide highly consistent genotyping results for blastocysts when compared to established methodologies [32], and the data's parallel nature can also be used to provide additional sequence information, as demonstrated here for the GBA mutations.

## Supporting Information

Figure S1 **ABI PRISM® 7900 HT Sequence Detection System Validation.** Allelic discrimination plots depicting the validation of the remaining assays on the ABI PRISM® 7900 HT Sequence Detection System. Water was used as the no template control.
(TIF)

Figure S2 **Applied Biosystems ViiA™ 7 Real-Time PCR System Validation.** Allelic discrimination plots depicting the validation of the remaining assays on the Applied Biosystems ViiA™ 7 Real-Time PCR System. Water was used as the no template control.
(TIF)

Figure S3 **Blind Validation.** Allelic discrimination plots depicting the blind results for the remaining assays on the ABI PRISM® 7900 HT Sequence Detection System. Water was used as the no template control.
(TIF)

Figure S4 **Original vs. redesigned assay results for the c.2207_2212delinsTAGATTC mutation.** Allelic discrimination plots showing the results for both the original and redesigned assay used to genotype the c.2207_2212delinsTAGATTC mutation. Separation between the wild type and heterozygous carrier cluster improved significantly for the redesigned assay.
(TIF)

Table S1 **Context sequences for the additional mutations used to design the TaqMan genotyping assays.**
(DOC)
