# Supplementary figures and images for: High-Throughput Carrier Screening Using TaqMan Allelic Discrimination
Source: PLoS One. 2013 Mar 26;8(3):e59722. doi: 10.1371/journal.pone.0059722 (PMC3608587; doi:10.1371/journal.pone.0059722)

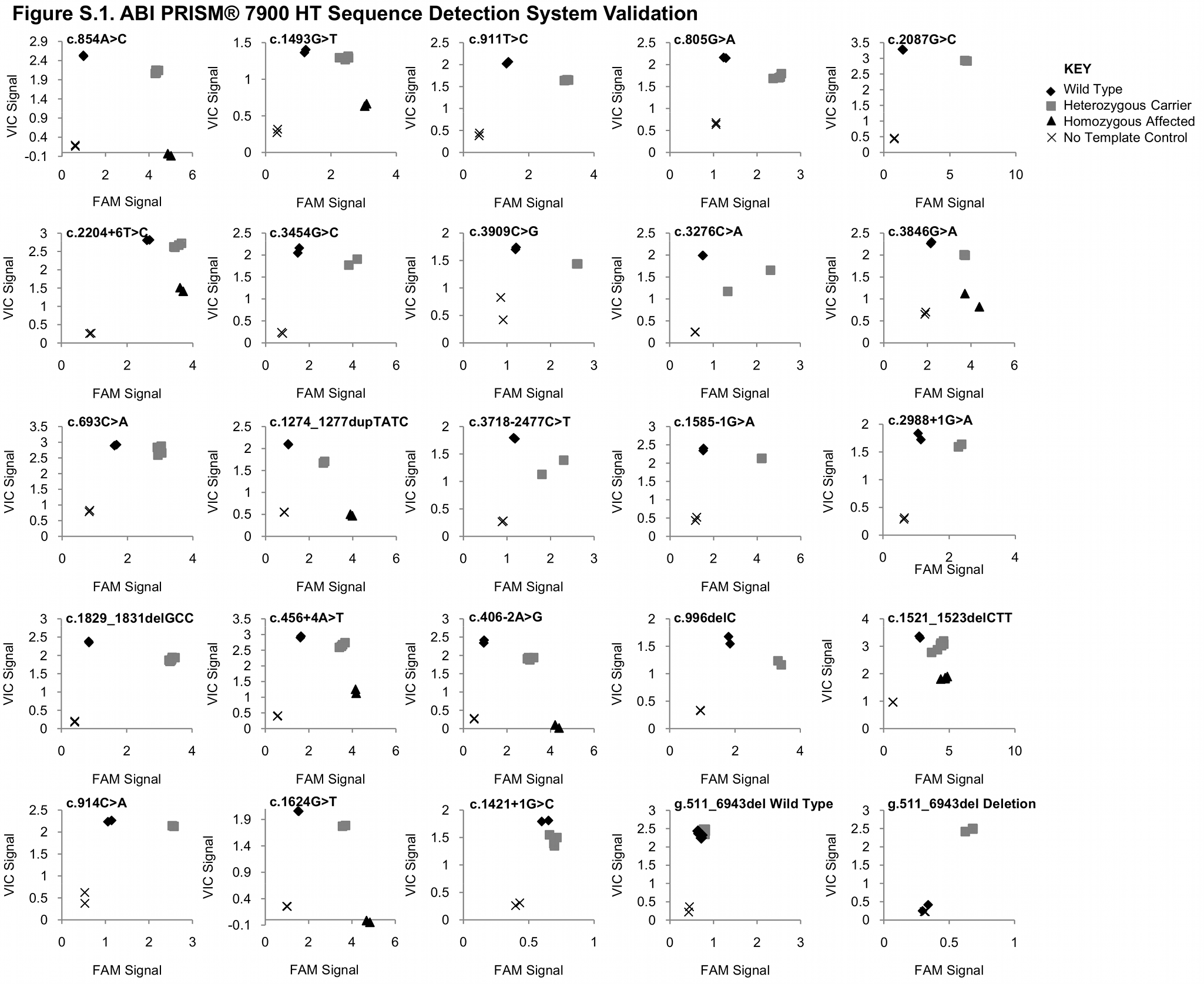

Supplement: Figure S1 — ABI PRISM® 7900 HT Sequence Detection System Validation. Allelic discrimination plots depicting the validation of the remaining assays on the ABI PRISM® 7900 HT Sequence Detection System. Water was used as the no template control. (TIF) [file pone.0059722.s001.tif]

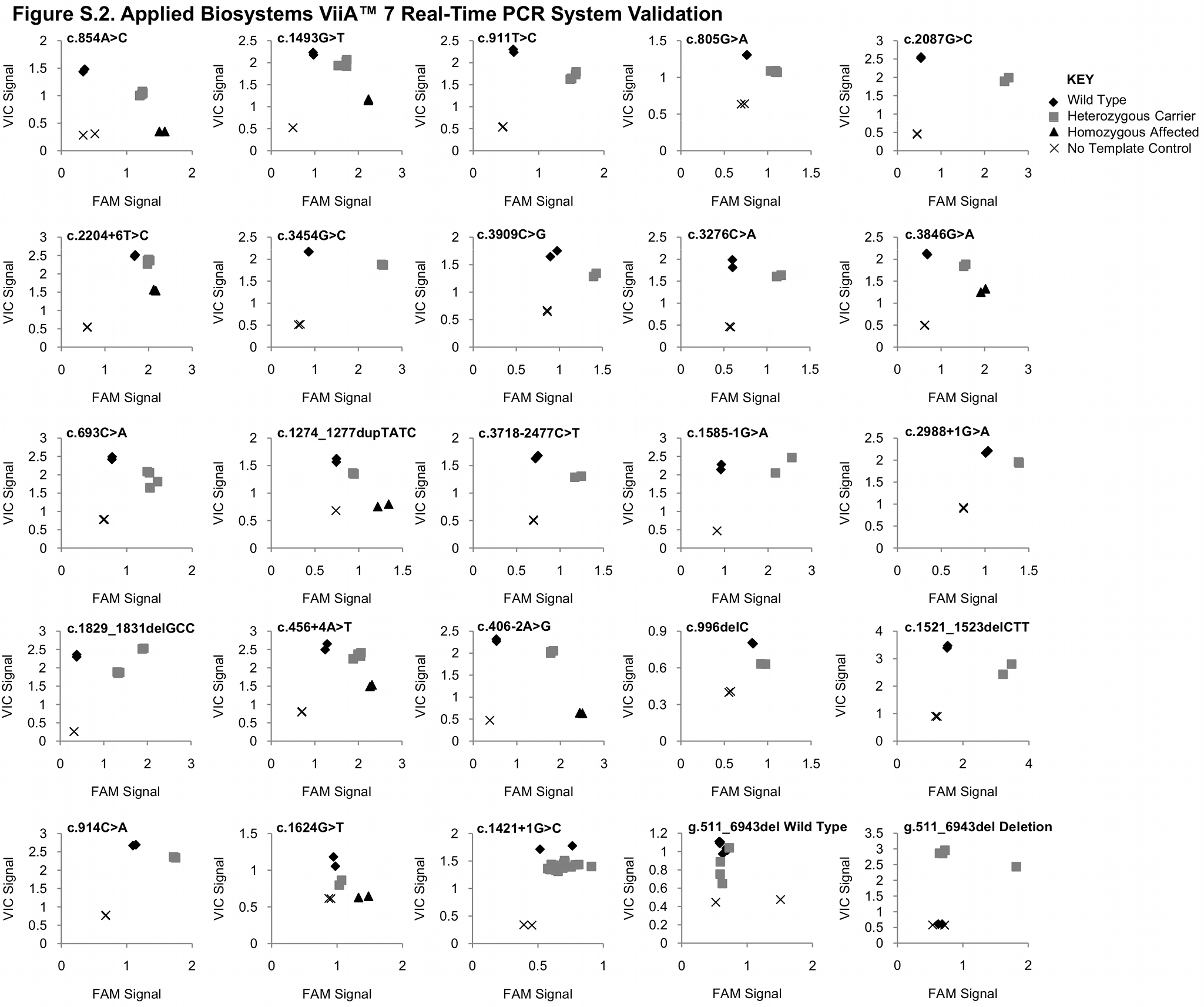

Supplement: Figure S2 — Applied Biosystems ViiA™ 7 Real-Time PCR System Validation. Allelic discrimination plots depicting the validation of the remaining assays on the Applied Biosystems ViiA™ 7 Real-Time PCR System. Water was used as the no template control. (TIF) [file pone.0059722.s002.tif]

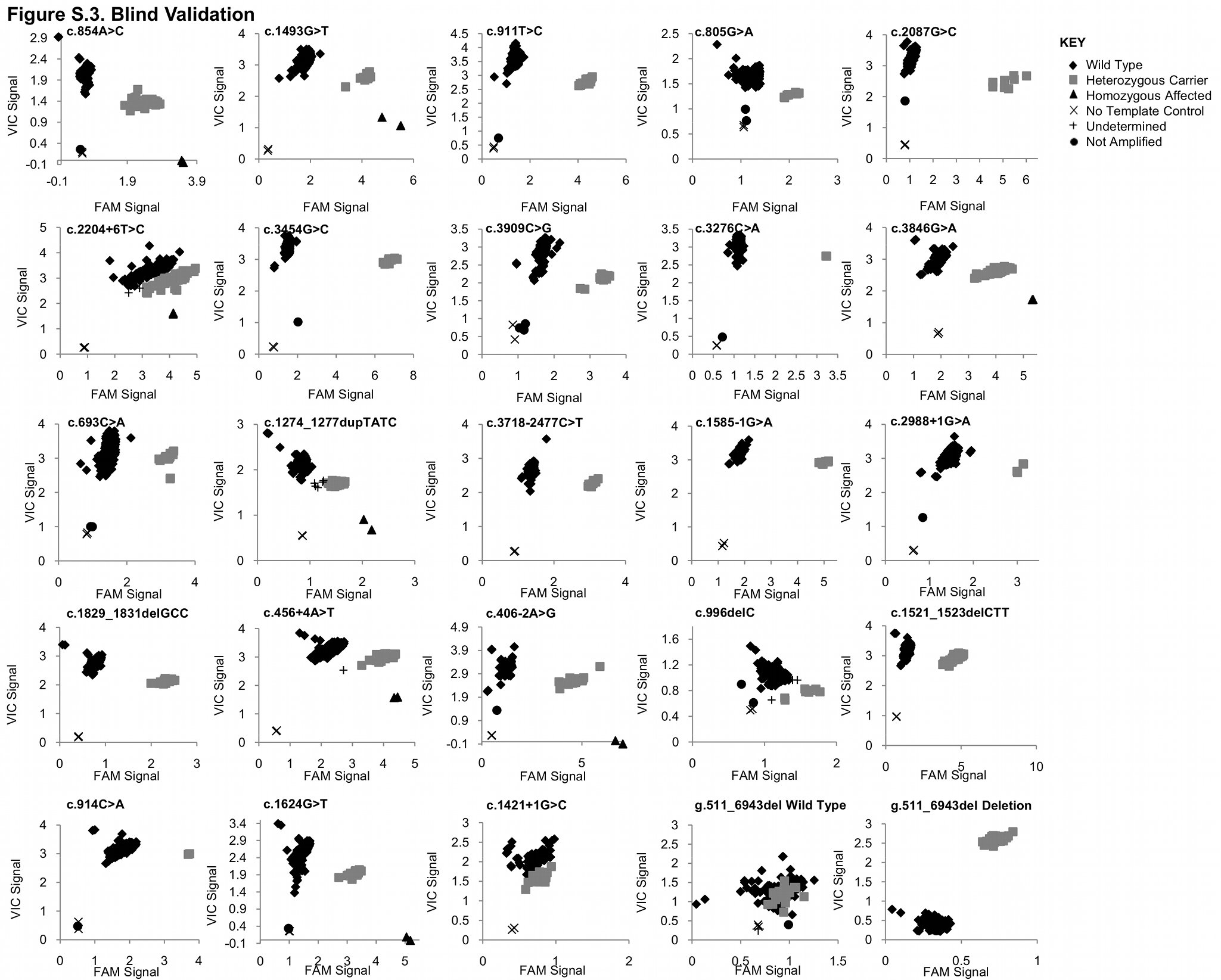

Supplement: Figure S3 — Blind Validation. Allelic discrimination plots depicting the blind results for the remaining assays on the ABI PRISM® 7900 HT Sequence Detection System. Water was used as the no template control. (TIF) [file pone.0059722.s003.tif]

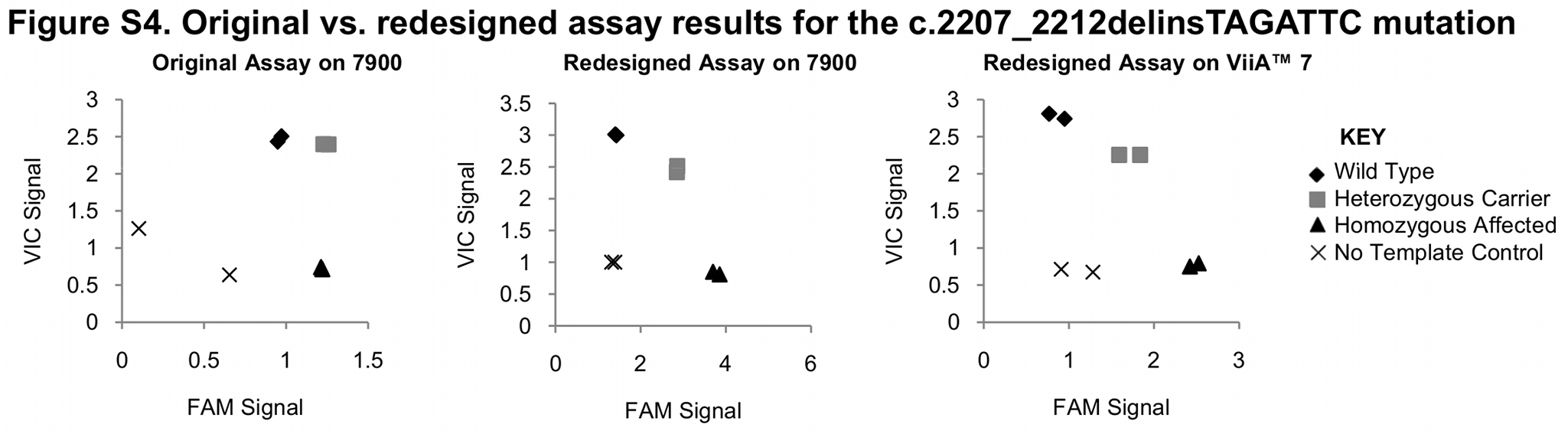

Supplement: Figure S4 — Original vs. redesigned assay results for the c.2207_2212delinsTAGATTC mutation. Allelic discrimination plots showing the results for both the original and redesigned assay used to genotype the c.2207_2212delinsTAGATTC mutation. Separation between the wild type and heterozygous carrier cluster improved significantly for the redesigned assay. (TIF) [file pone.0059722.s004.tif]
