# Supplementary material for: High-Throughput Carrier Screening Using TaqMan Allelic Discrimination
Source: PLoS One. 2013 Mar 26;8(3):e59722. doi: 10.1371/journal.pone.0059722 (PMC3608587; doi:10.1371/journal.pone.0059722)
Supplement: Table S1 — Context sequences for the additional mutations used to design the TaqMan genotyping assays. (DOC) [file pone.0059722.s005.doc]

Supplementary Table 1. Context sequences for the additional mutations used to design the TaqMan genotyping assays.

| Mutation | Context Sequence |
| --- | --- |
| c.2207_2212delinsTAGATTC | TTTATAAAACCTAAGGACAAATGTAATTTTGTCAGGTTAATGTATAAAATTGAAATTGTTTACTACTTTTATACTTAGATTCCAGCTACAT**[ATCTGA/TAGATTC]**CAGGTGATAAGACTGACTCAGAAGCTACAAATATTTACCTCCAGTTATCAAAAAAAGACCCAATCATAAAACTTCTATAT |
| c.914C>A | AGACTGTACCGTGTACCCCGTGTTTGTGAATGAGGCCGCATATTACGAAAAGAAAGAAGCTTTTGCAAAGACAACTAAACTAACGCTCAATG**[C/A]**AAAAAGTATTCGCTGCTGTTTACATTAGAAATCACTTCCAGCTTACATCTTACACGGTGTCTTACAAATTCTGCTAGTCTGTAAGCTCCT |
| c.854A>C | ATCCTGGGGATCCCATGTTTTTAACTCTTGATGGGAAGACGATCCCACTGGGCGGAGACTGTACCGTGTACCCCGTGTTTGTGAATG**[A/C]**GGCCGCATATTACGAAAAGAAAGAAGCTTTTGCAAAGACAACTAAACTAACGCTCAATGCAAAAAGTATTCGCTGCTGTTTACATTAGAAATCAC |
| c.693C>A | CCGGCCCAGAGATGTTTTTAGTTGCCATTGATACATATTGTTTTTGTCATAGGAAAAGAATTTCCTCCCTGCGCCATTGAGGTCTATAAAATTATAGAGAAAGTTGATTA**[C/A]**CCCCGGGATGAAAATGGAGAAATTGCTGCTATCATCCATCCTAATCTGCAGGTAACATTTGTTCTTTCTTTA |
| c.3454G>C | TGGTATTATCCTGACTTTAGCCATGAATATCATGAGTACATTGCAGTGGGCTGTAAACTCCAGCATA**[G/C]**ATGTGGATAGCTTGGTAAGTCTTATCATCTTTTTAACTTTTATGAAAAAAATTCAGACAAGTAACAAAGTATGAGTAATAGCATGAGGAAGAACTATATACCGTATATTGAGCTT |
| c.3909C>G | GAAAAATAAAAAGTTATTTAAGTTATTCATACTTTCTTCTTCTTTTCTTTTTTGCTATAGAAAGTATTTATTTTTTCTGGAACATTTAGAAAAAA**[C/G]**TTGGATCCCTATGAACAGTGGAGTGATCAAGAAATATGGAAAGTTGCAGATGAGGTAAGGCTGCTAACTGAAATGATTTTGAAAGGG |
| c.3276C*>*A | TAAAAGGACTATGGACACTTCGTGCCTTCGGACGGCAGCCTTACTTTGAAACTCTGTTCCACAAAGCTCTGAATTTACATACTGCCAACTGGTTCTTGTA**[C/G]**CTGTCAACACTGCGCTGGTTCCAAATGAGAATAGAAATGATTTTTGTCATCTTCTTCATTGCTGTTACCTTCATTTCCATTT |
| c.3846G>A | ACTGGATCAGGGAAGAGTACTTTGTTATCAGCTTTTTTGAGACTACTGAACACTGAAGGAGAAATCCAGATCGATGGTGTGTCTTGGGATTCAATAACTTTGCAACAGTG**[G/A]**AGGAAAGCCTTTGGAGTGATACCACAGGTGAGCAAAAGGACTTAGCCAGAAAAAAGGCAACTAAATTATATT |
| c.1624G>T | CAGATTGAGCATACTAAAAGTGACTCTCTAATTTTCTATTTTTGGTAATAGGACATCTCCAAGTTTGCAGAGAAAGACAATATAGTTCTT**[G/T]**GAGAAGGTGGAATCACACTGAGTGGAGGTCAACGAGCAAGAATTTCTTTAGCAAGGTGAATAACTAATTATTGGTCTAGCAAGCATTTGCTG |
| c.1521 1523delCTT | GAACTGGAGCCTTCAGAGGGTAAAATTAAGCACAGTGGAAGAATTTCATTCTGTTCTCAGTTTTCCTGGATTATGCCTGGCACCATTAAAGAAAATATCAT**[CTT/-]**TGGTGTTTCCTATGATGAATATAGATACAGAAGCGTCATCAAAGCATGCCAACTAGAAGAGGTAAGAAACTATGTGAAA |
| c.3718-2477C>T | TGGATCTAAATTTCAGTTGACTTGTCATCTTGATTTCTGGAGACCACAAGGTAATGAAAAATAATTACAAGAGTCTTCCATCTGTTGCAGTATTAAAATGG**[C/T]**GAGTAAGACACCCTGAAAGGAAATGTTCTATTCATGGTACAATGCAATTACAGCTAGCACCAAATTCAACACTGTTTAACT |
| c.1585-1G>A | TGTGGTTAAAGCAATAGTGTGATATATGATTACATTAGAAGGAAGATGTGCCTTTCAAATTCAGATTGAGCATACTAAAAGTGACTCTCTAATTTTCTATTTTTGGTAATA**[G/A]**GACATCTCCAAGTTTGCAGAGAAAGACAATATAGTTCTTGGAGAAGGTGG AATCACACTGAGTGGAGGTCA |
| c*.*2988+1G*>*A | TAAAGTATGCAAAAAAAAAAAAAGAAATAAATCACTGACACACTTTGTCCACTTTGCAATGTGAAAATGTTTACTCACCAACATGTTTTCTTTGATCTTACA**[G/A]**TTGTTATTAATTGTGATTGGAGCTATAGCAGTTGTCGCAGTTTTACAACCCTACATCTTTGTTGCAACAGTGCCAGTGAT |
| c.2087G>C | GTGTTTAGCATTACAGGCCGGCCTGAGCAGCAATCATGTGTCCCATGGGGAAGTTCTGCGGAAAGTGGAGAGGGGTTCAC**[G/C]**GATTGTCACTGTTGTGCCCCAGGACACAAAGCTTGTATTACAGGTAAGCTGGTTTTTCAGACAAGATAGATAGTCTGATTGTCATTCAGCCAAGTACCAAGC |
| c.2204+6T>C | CCAAGGGGAAACTTAGAAGTTGTTCATCATCGAGCCCTGGTTTTAGCTCAGATTCGGAAGTGGTTGGACAAGTAAG**[T/C]**GCCATTGTACTGTTTGCGACTAGTTAGCTTGTGATTTATGTGTGAAGACAATAAGTATTTTATTACAATTTCGAGAACTTAAAATTATGAAAAGCCCTCATTACCT |
| c.456+4A>T | CAAGGTCTTGGGTATGCACCTATAGATTACTATCCTGGTTTGCTTAAAAATGTG**[A/T]**GTATTTAAAATTTATCACTTTTGAAATGTTTAATGCTGAATGTGCCATCAGCAAAAAGAGTAAATGGAAATATTTCAGTCCTCCAGAAGAGATGTTTAACTTTTCTTTGTTTATCTCTTCTTACCTTG |
| c.1493G>T | TGTGGCCCCTCCCTGGAGTTACCCTTGCTCCTTGCCCCTCCAGTCAGCCCCACATCCTTGCAGGTTACC**[G/T]**TGTGTACCAAATAGATGGAAACTACTCCGGGAGCTCTCACGTGGTCCTGGACCATGAGACCTACATCCTGAATCTGACCCAGGCAAACATACCGGGAGCCATACCGCACTGGC |
| c.911T>C | TATGGTGTACTGGACAGGAGACATCCCCGCACATGATGTCTGGCACCAGACTCGTCAGGACCAACTGCGGGCCCTGACCACCGTCACAGCAC**[T/C]**TGTGAGGAAGTTCCTGGGGCCAGTGCCAGTGTACCCTGCTGTGGGTAACCATGAAAGCACACCTGTCAATAGCTTCCCTCCCCCCTTCAT |
| c.996delC | CTTGTGAGGAAGTTCCTGGGGCCAGTGCCAGTGTACCCTGCTGTGGGTAACCATGAAAGCACACCTGTCAATAGCTTCCCTCCCCC**[C/-]**TTCATTGAGGGCAACCACTCCTCCCGCTGGCTCTATGAAGCGATGGCCAAGGCTTGGGAGCCCTGGCTGCCTGCCGAAGCCCTGCGCACCCTCAGG |
| delR608 | ACCCTCGGAGCCCTGTGGCACGCCCTGCCGTCTGGCTACTCTTTGTGCCCAGCTCTCTGCCCGTGCTGACAGCCCTGCTCTGTGC**[CGC/-]**CACCTGATGCCAGATGGGAGCCTCCCAGAGGCCCAGAGCCTGTGGCCAAGGCCACTGTTTTGCTAGGGCCCCAGGGCCCACATTTGGGAAAGTTC |
| c.1274_1277dupTATC | AACTATATGAAGGAGCTGGAACTGGTCACCAAGGCCGGCTTCCGGGCCCTTCTCTCTGCCCCCTGGTACCTGAACCGTATATC**[*/TATC]**CTATGGCCCTGACTGGAAGGATTTCTACGTAGTGGAACCCCTGGCATTTGAAGGTGAAAGCAGAGAGCTCTCCTTGCTAACCAAAGGAGGCTGGGT |
| c.805G>A | TCATTGAATACGCACGGCTCCGGGGTATCCGTGTGCTTGCAGAGTTTGACACTCCTGGCCACACTTTGTCCTGGGGACCA**[G/A]**GTAAGAATGATGTCTGGGACCAGAGGGACTCTGCTTGTTATGCTCAGAGTGAAGCTTCAGGGCACTGGCTCATGGAAGTGGCATATCCCAGCCTTGGTCCTT |
| c.1421+1G>C | AGCAGAAGGCTCTGGTGATTGGTGGAGAGGCTTGTATGTGGGGAGAATATGTGGACAACACAAACCTGGTCCCCAGGCTCTG**[G/A]**TAAGGGTTTTCGGGGGGGAGGTGGAGGGTTGGGCCTGAGAGCAGGAGTTCTCCCTTAGAGACCCAATCCCATCTAGCCACCTCTGGACTAGTACCCTTCT |
| c.406-2A>G | GGGCGGGCAGGTGCTGGTGGGCGGGCAGGTGCAGGTGGGTGGGCTGCAGAGAGCGGGCCGGACTCACAGGCCCTCCCCTTCTCTGCCCAC**[A/G]**GTACCTGGCGTTGCCTGACGTGTCACTGGGCCGGTATGCGTATGTCCGTGGTGGGGGTGACCCTTGGACCAATGGCTCAGGGCTTGCTCTCT |
| g.511_6943del wild type | CCACCTGTCATGTGGACCTTGGGGCTTGGGGCTGCCAAGGTTTACTCTGCCCCCAACTGGCCCCCACAGATCACGTTTGA**|**CAACAAAGCACACAGTGGGCGGATCCCCATCAGCCTGGAGACCCAGGCCCACATCCAGGAGTGTAAGCACCCCAGTGTCTTCCAGCACGGTGAGCCCCTGAGCCCC |
| g.511_6943del deletion | TTTTTTTTAGACAGTCTTGCTCTGTTGCCCAGGCTGGAGTGCAGTGGTGTGATCATAGCTCACTGCAGCCTCGACCTCCTGGGCT**|**CAACAAAGCACACAGTGGGCGGATCCCCATCAGCCTGGAGACCCAGGCCCACATCCAGGAGTGTAAGCACCCCAGTGTCTTCCAGCACGGTGAGCCCCTGA |

*Bold font and brackets indicate the mutation sites.

^†^ The break point for the large deletion assays are indicated by a line.
